# Identification and Quantitative Characterization of PSORI-CM01, a Chinese Medicine Formula for Psoriasis Therapy, by Liquid Chromatography Coupled with an LTQ Orbitrap Mass Spectrometer

**DOI:** 10.3390/molecules20011594

**Published:** 2015-01-19

**Authors:** Shao-Dan Chen, Chuan-Jian Lu, Rui-Zhi Zhao

**Affiliations:** 1 The Second Clinical College, Guangzhou University of Chinese Medicine, Guangzhou 510000, China; E-Mails: shaodanchen@126.com (S.-D.C.); 13610241754@163.com (R.-Z.Z.); 2 Guangdong Provincial Hospital of Chinese Medicine, Guangzhou 510000, China; 3 Postdoctoral Programme, Guangzhou University of Chinese Medicine, Guangzhou 510000, China

**Keywords:** PSORI-CM01, UHPLC-ESI-LTQ/Orbitrap-MS, identification, quantification, TCM

## Abstract

PSORI-CM01 is a Chinese medicine formula prepared from medicinal herbs and used in China for the treatment of psoriasis. However, the chemical constituents in PSORI-CM01 have not been clarified yet. In order to quickly define the chemical profiles and control the quality of PSORI-CM01 preparations, ultra-high liquid chromatography coupled with electrospray ionization hybrid linear trap quadrupole Orbitrap mass spectrometry (UHPLC-ESI-LTQ/Orbitrap-MS) was applied for simultaneous identification and quantification of multiple constituents. A total of 108 compounds, including organic acids, phenolic acids, flavonoids, and terpenoids, were identified or tentatively deduced on the base of their retention behaviors, MS and MS^n^ data, or by comparing with reference substances and literature data. In addition, an optimized UHPLC-ESI-MS method was established for the quantitative determination of 14 marker compounds in different dosage forms of PSORI-CM01 preparations. The validation of the method, including spike recoveries, linearity, sensitivity (LOQ), precision, and repeatability, was carried out and demonstrated to be satisfied the requirements of quantitative analysis. This is the first report on the comprehensive determination of chemical constituents in PSORI-CM01 preparations by UHPLC-ESI-LTQ/Orbitrap mass spectrometry. The results suggested that the established methods would be a powerful and reliable analytical tool for the characterization of multi-constituents in complex chemical system and quality control of TCM preparations.

## 1. Introduction

As known, the Chinese nation, with a history of more than 5000 years of civilization, largely relies on Traditional Chinese Medicine (TCM). TCM still plays a huge role in health care, disease prevention and treatment in China today. This is the reason why TCM has been deeply rooted in China and other Chinese cultural circles around the world for thousands of years. Traditional medicine, especially TCM, is not only an important and indispensable “alternative medicine” or “complementary medicine”, but has also been entrusted with hopes for disease prevention and treatment in the future.

Traditional Chinese medicine prescriptions (TCMPs), or TCM formulae, which are applied according to certain compatibility rules, are the main and important clinical applications of TCM. However, TCMPs are facing difficulties because their effective constituents are unclear and sometimes inexplicable, which seriously restricts their development in the international market [1]. The chemical constituents of TCMPs are the key object of the study of TCM [2]. TCM is commonly considered to operate due to the synergistic effects of all the major and minor components in the medicines. Hence sensitive and comprehensive analytical techniques are needed to acquire a better understanding of the substance basis of TCM and to enhance the product quality control [3].

PSORI-CM01 was a novel formulated Chinese medicine used for psoriasis therapy [4]. It was optimized on the basis of a Chinese medicine formula Yin-Xie Ling, which was originated by Guo-Wei Xuan, the State Medical Master of China [5,6]. PSORI-CM01 was composed of seven herbs including *Rhizoma Curcumae* (E Zhu), *Radix Paeoniae Rubra* (Chi Shao), *Sarcandra glabra* (Zhong-Jie Feng), *Radix Arnebiae* (Zi Cao), *Rhizoma Smilacis Glabrae* (Tu-Fu Ling), *Fructus Mume* (Wu Mei), and *Radix Glycyrrhizae* (Gan Cao). Guo-Wei Xuan believed that one of the main causes of psoriasis is blood stasis. In this TCM formula PSORI-CM01, *Curcumae* bearing blood-activating and stasis-dissolving efficacy acts as the monarch drug, *Paeoniae Rubra*, *Sarcandra glabra* and *Arnebiae*, as the minister drugs, that together help *Curcumae* in activating blood and removing blood stasis. *Smilacis Glabrae* and *Mume* produce the effect of reducing the itch and together act as assistant drugs. *Licorice*, as a guide drug can mediate the other drugs’ properties. When combined, the seven drugs have great therapeutic effects. Although the chemical constituents of the individual herbs have previously been well studied [7,8,9,10,11,12,13,14,15], little is known about the integrated chemical composition of PSORI-CM01. Unlike chemical drugs, botanical products contain a complex mixture of compounds. The contents of these compounds may be significantly affected by plant species, geographical sources, harvesting, processing and storage [16]. In addition, three dosage forms of PSORI-CM01 preparations (tablet, granules and decoction) are produced and used clinically in the Guangdong Provincial Hospital of Chinese Medicine. In order to guarantee drug safety and batch-to-batch consistency, quality control is therefore critically important for preparations such as PSORI-CM01. In recent years, the combination of Orbitrap technology with a linear ion trap, known as LTQ Orbitrap mass spectrometer were introduced, which could provide all the traditional MS and MS^n^ scan functions using a linear IT and high mass accuracy measurements (errors within 5 ppm) [17]. Our previous study indicated that this analytical technique has the potential capability of simple, sensitive and reliable detection and identification of complex samples such as TCMs [9].

In the present study, a sensitive LC-ESI-MS^n^ method was established for rapid separating, reliable identifying and quantifying the multiple components in PSORI-CM01 preparations, by using a hybrid LTQ-Orbitrap mass spectrometer coupled with an UHPLC system. The qualitative analysis was carried out in negative ionization mode to acquire accurate mass data in full scan mode and MS/MS in a data dependent product ion spectrum. Further, 14 reference compounds were quantitatively determined in negative ionization mode and eight samples of PSORI-CM01 preparations were analyzed for assessment of quality consistence.

## 2. Results and Discussion

### 2.1. Optimization of Chromatographic Conditions

To improve the resolution and sensitivity of the analysis but reduce the analytical time, the mobile phase system was optimized. To inhibit ionization of the acidic ingredients in PSORI-CM01, formic acid was added to the mobile phase. Two mobile phase systems, methanol-aqueous solution and acetonitrile-aqueous solution were compared. Both negative and positive modes were examined. Generally, in positive mode, low abundance of [M+H]^+^, [M+NH_4_]^+^ ions and few product ions were observed, while, in negative ion mode, a series of [M−H]^−^ ions and/or adduct ions ([M+HCOOH−H]^−^) appeared with sufficient abundance. Thus the negative ion mode was chosen and the [M−H]^−^/([M+HCOOH−H]^−^) ions were further subjected to LC-MS^n^ analysis. For the extracted target ions in full scan mode, an accurate mass limit of 5 ppm accurate mass filter limit was used to characterize “real” compounds signals from the background peaks, as well as to increase the signal-to-noise ratio for each analyte.

### 2.2. Identification of Chemical Constituents in PSORI-CM01 Preparations

**Figure 1:**
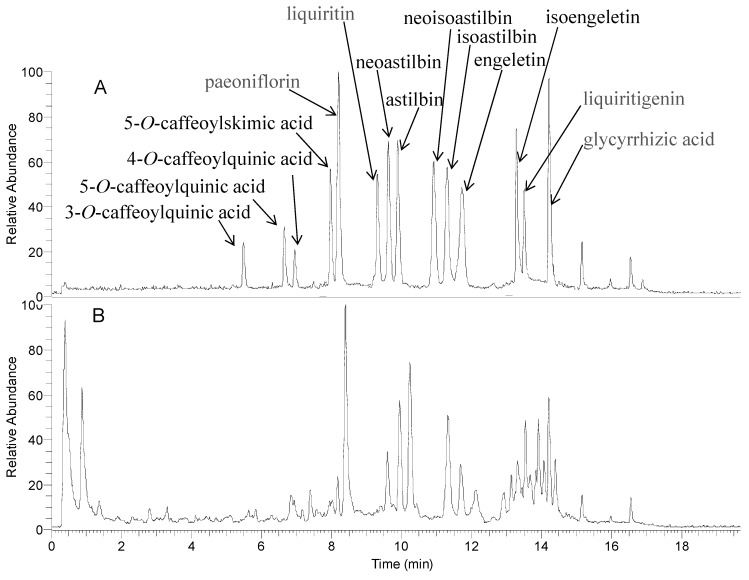
UHPLC-(-) ESI-MS total ion chromatograms of a mixture of fourteen standards (**A**) and PSORI-CM01 (**B**).

The reference substances and PSORI-CM01 sample were analyzed by using the optimized LC-ESI-MS^n^ method. The TIC chromatogram of PSORI-CM01 sample in negative ESI mode was shown in Figure 1, and 108 peaks were observed in PSORI-CM01 sample. The MS data showed high precision with all the mass accuracy within 5 ppm. For most of the constituents, [M−H]^−^ ions were observed. Due to the use of formic acid in mobile phase, there were adduct ions of [M+46−H]^−^ corresponding to [M+HCOOH−H]^−^ in negative ion mode. These results provided valuable information for confirming accurate molecular weight and composition of the constituents.

One hundred and eight compounds were tentatively identified on the basis of their retention behaviors, accurate molecular weights and MS^n^ fragment data, or by comparison with reference substances or literature data. Corresponding quasimolecular ions and their fragment ions in the MS^n^ spectra were listed in Table 1. The identified compounds can be classified into four classes including organic acids, phenolic acids, flavonoids, and terpenoids, which were mainly from *Radix Paeoniae Rubra*, *Rhizoma Smilacis Glabrae*, *Sarcandra glabra,* and *Radix Glycyrrhizae.* Monoterpene glucosides and phenolic acids are the main constituents of *Paeoniae Rubra*, caffeic acid derivatives and flavonoids are abundant in *Sarcandra glabra*, flavonoids and phenolic compounds are mainly from *Rhizoma Smilacis Glabrae*, while flavonoids and triterpene glucoside are from *Radix Glycyrrhizae.* To some extent, the UHPLC-ESI-MS chromatogram reflected the rationale of PSORI-CM01. However, the characteristic constituents (sesquiterpenoids and curcuminoids) from the monarch drug *Rhizoma Curcumae* were not detected.

Among the 108 compounds, 3-*O*-caffeoylquinic acid (**16**), 5-*O*-caffeoylquinic acid (**22**), 4-*O*-caffeoylquinic acid (**24**), 5-*O*-caffeoylskimic acid (**39**), paeoniflorin (**40**), liquiritin (**51**), neoastilbin (**56**), astilbin (**60**), neoisoastilbin (**64**), isoastilbin (**67**), engeletin (**79**), isoengeletin (**82**), liquiritigenin (**87**) and glycyrrhizic acid (**107**) were the main components in PSORI-CM01. Besides, 3-*O*-caffeoylquinic acid (**16**), paeoniflorin (**40**), liquiritin (**51**), astilbin (**60**), engeletin (**79**), liquiritigenin (**87**) and glycyrrhizic acid (**107**) have been reported to have multiple biological activities such as anti-inflammation, immunoregulation and anti-tumor properties [18,19,20,21,22,23], which are related to psoriasis. Thus, these 14 ingredients were selected as markers for quality control.

**Table 1:** Identification of the chemical constituents of PSORI-CM01 by UHPLC-ESI-MS^n^ analysis.

Peak No.	*t*R (min)	SelectedIon	ObservedMass (*m/z*)	CalculatedMass (*m/z*)	Formula	MS/MS PatternsFragmentation	Identifieation	Source ^a^	Reference
1	0.41	[M−H]	191.0559	191.0556	C_7_H_12_O_6_	191→173, 127, 109	quinic acid	Sa	[8]
2	0.51	[M−H]	133.0140	133.0137	C_4_H_6_O_5_	133→115, 87, 71	malic acid	M	
3	0.60	[M−H]	173.0451	173.0450	C_7_H_10_O_5_	173→155, 145, 129	shikimic acid	Sm	[9]
4	0.88	[M−H]	191.0198	191.0192	C_6_H_8_O_7_	191→173, 155→111	citric acid	M	
5	0.98	[M−H]	115.0037	115.0031	C_4_H_4_O_4_	115→97, 71	fumaric acid	Sa	[8]
6	2.16	[M−H]	169.0142	169.0137	C_7_H_6_O_5_	169→125	gallic acid	P	[24,25]
7	2.60	[M−H]	331.0663	331.0665	C_13_H_16_O_10_	331→169	gallic acid-1-*O*-glucoside	P	
8	3.28	[M−H]	315.0716	315.0716	C_13_H_16_O_9_	315→297, 247,153	protocatechuic acid-4-*O*-glucoside	P, C	
9	3.64	[M−H]	197.0453	197.0450	C_9_H_10_O_5_	197→153, 123	syringic acid	Sa, Sm	[9]
10	3.69	[M−H]	315.0716	315.0716	C_13_H_16_O_9_	315→169	gallic acid-1-*O*-rhamnoside	P	
11	3.78	[M−H]	153.0192	153.0188	C_7_H_6_O_4_	153→109	protocatechuic acid	Sa,Sm,C	[8,9]
12	3.82	[M−H]	493.1208	493.1193	C_19_H_26_O_15_	493→313, 169	gallic acid-1-*O*-glucosyl-(6→1)-glucoside	P	
13	4.02	[M−H]	359.0977	359.0978	C_15_H_20_O_10_	359→197, 179	syringic acid-4-*O*-glucoside	Sa, Sm	[9]
14	4.83	[M−H]	181.0495	181.0450	C_9_H_10_O_5_	197→169, 133	3,5-dimethoxy-4-hydroxy-benzaldehyde	Sa	
15	4.93	[M−H]	137.0243	137.0239	C_7_H_6_O_3_	137→109, 93	4-hydroxybenzoic acid	C, M, Sm, Sa,	
16	5.01	[M−H]	353.0873	353.0873	C_16_H_18_O_9_	353→191, 179→135	3-*O*-caffeoylquinic acid	Sa	[8]
17	5.24	[M−H]	165.0556	165.0552	C_9_H_10_O_3_	165→151, 135	paeonal	P	[24,25]
18	5.31	[M−H]	345.1182	345.1186	C_15_H_22_O_9_	345→183	(3,5-dimethoxy-4-hydroxyphenyl)methy-*O*-glucoside	Sa	
19	5.59	[M−H]	183.0296	183.0293	C_8_H_8_O_5_	183→124	methyl gallate	P	
20	6.20	[M−H]	609.1445	609.1456	C_27_H_30_O_16_	609→445, 301	rutin	M	
21	6.20	[M−H]	495.1498	495.1503	C_23_H_28_O_12_	495→465,333,281	oxypaeoniflorin	P	
22	6.12	[M−H]	353.0872	353.0873	C_16_H_18_O_9_	353→191, 179, 135	5-*O*-caffeoylquinic acid	Sa	[8]
23	6.17	[M−H]	289.0713	289.0712	C_15_H_14_O_6_	289→245, 205, 179	(+)-catechin	P, Sm	[9]
24	6.18	[M−H]	335.0767	335.0767	C_16_H_16_O_8_	335→289, 179, 135, 111	3-*O*-caffeoylshikimic acid	Sa, Sm	[9]
25	6.21	[M−H]	321.0246	321.0247	C_14_H_10_O_9_	321→169	*p*-digallic acid	P	
26	6.41	[M−H]	353.0873	353.0873	C_16_H_18_O_9_	353→191, 179, 135	4-*O*-caffeoylquinic acid	Sa	[8]
27	6.43	[M−H]	469.1129	469.1135	C_24_H_22_O_10_	469→423, 371, 315, 289	8-[β-(3,4-dihydroxyphenyl)-α-carboxyl-3-xoxpropyl]-substituted catechin	Sm	[9]
28	6.45	[M−H]	179.0345	179.0344	C_9_H_8_O_4_	179→135, 85	caffeic acid	Sa, Sm, A	[8,9]
29	6.59	[M+COOH]	447.1500	447.1503	C_18_H_26_O_10_	447→401, 349, 317, 191	phenylmethyl-glucoside-(6→1)-apiose	M	
30	6.62	[M−H]	221.0452	221.0450	C_11_H_10_O_5_	221→206	fraxidin	Sa	[8]
31	6.67	[M−H]	369.0821	369.0822	C_16_H_18_O_10_	369→207	fraxin	Sa	[8]
32	6.87	[M−H]	435.1289	435.1291	C_21_H_24_O_10_	435→273	isoliquiritigenin-7-*O*-glucoside	Sa, P	[8]
33	6.87	[M−H]	431.1914	431.1917	C_20_H_32_O_10_	431→385, 223, 205, 153	drovomifoliol-*O*-glucopyranoside	Sa	[8]
34	6.96	[M−H]	433.2070	433.2074	C_20_H_34_O_10_	433→387, 369 , 225	dihydrovomifoliol-*O*-glucoside	Sa	[8]
35	7.13	[M−H]	335.0767	335.0767	C_16_H_16_O_8_	335→289, 179, 135, 111	4-*O*-caffeoylshikimic acid	Sa, Sm	[9]
36	7.20	[M−H]	289.0712	289.0712	C_15_H_14_O_6_	289→245, 205, 179	epi-catechin	P, Sm	[9]
37	7.23	[M+COOH]	525.1604	481.1710	C_23_H_30_O_11_	525→479, 465, 121	albiflorin	P	[24]
38	7.28	[M−H]	207.0296	207.0293	C_10_H_8_O_5_	207→192	fraxetin	Sa	[8]
39	7.38	[M−H]	335.0767	335.0767	C_16_H_16_O_8_	335→289, 179, 135, 111	5-*O*-caffeoylshikimic acid	Sa, Sm	[9]
40	7.61	[M+COOH]	525.1604	481.1710	C_23_H_30_O_11_	525→479, 327, 283,161	paeoniflorin	P	[24,25]
41	7.72	[M−H]	471.1863	471.1866	C_22_H_32_O_11_	471→425, 263	sarcaglaboside G	Sa	[8]
42	7.81	[M−H]	339.0715	339.0716	C_15_H_16_O_9_	339→193, 165,137	6,7,8-trihydroxycoumarin-7-rhamnoside	Sa, Sm	[9]
43	7.94	[M−H]	629.1514	629.1506	C_30_H_30_O_15_	629→483, 475, 449, 303, 285	8-[β-(3,4-dihydroxyphenyl)-α-carboxyl-3-oxopropyl]-substituted neoastilbin	Sm	[9]
44	8.05	[M−H]	473.2022	473.2023	C_22_H_34_O_11_	473.202	sarcaglaboside H	Sa	[8]
45	8.16	[M−H]	629.1514	629.1506	C_30_H_30_O_15_	629→483, 475, 449, 303, 285	8-[β-(3,4-dihydroxyphenyl)-α-carboxyl-3-oxopropyl]-substituted astilbin	Sm	[9]
46	8.18	[M−H]	565.1552	565.1557	C_26_H_30_O_14_	565→313, 193	(2*R*/2*S*)-naringenin-6-*C*-β-d-glucopy ranoside-(6-1)-apiose	Sa	[8]
47	8.27	[M−H]	537.1025	537.1033	C_27_H_22_O_12_	537→493, 295, 159, 109	lithospermic acid	A	
48	8.33	[M−H]	565.1550	565.1557	C_26_H_30_O_14_	565→313, 193	(2*R*/2*S*)-naringenin-6-*C*-glucopyranoside-(6-1)-apiose	Sa	[9]
49	8.52	[M−H]	629.1514	629.1506	C_30_H_30_O_15_	629→483, 475, 449, 303, 285	8-[β-(3,4-dihydroxyphenyl)-α-carboxyl-3-oxopropyl]-substituted	Sm	[9]
50	8.57	[M−H]	221.0452	221.0450	C_11_H_10_O_5_	221→206, 191, 163	isofraxidin	Sa	[8]
51	8.62	[M−H]	417.1186	417.1186	C_21_H_22_O_9_	417→255	liquiritin	G	[26]
52	8.62	[M−H]	549.1607	549.1608	C_26_H_30_O_13_	549→429, 255	liquiritin apioside	G	[26]
53	8.77	[M−H]	451.1025	451.1029	C_24_H_20_O_9_	451→341, 299	cinchonain Ia	Sm	
54	8.80	[M−H]	303.0505	303.0505	C_15_H_12_O_7_	303→285	taxifolin	Sm	[9]
55	8.82	[M−H]	300.9986	300.9984	C_14_H_6_O_8_	301→257	gallogen	P	
56	8.84	[M−H]	449.1088	449.1084	C_21_H_22_O_11_	449→303, 285	neoastilbin	Sa, Sm	[8,9]
57	8.98	[M−H]	629.1514	629.1506	C_30_H_30_O_15_	629→483, 475, 449, 303, 285	8-[β-(3,4-dihydroxyphenyl)-α-carboxyl-3-oxopropyl]-substituted isoastilbin	Sm	[9]
58	8.96	[M−H]	631.1656	631.1663	C_30_H_32_O_15_	631→613, 491, 399, 169	galloyl paeoniflorin	P	
59	9.02	[M−H]	477.0668	477.0669	C_21_H_18_O_13_	477→301	quercetin-3-*O*-glucruronide	Sa	[8]
60	9.02	[M−H]	449.1088	449.1084	C_21_H_22_O_11_	449→303, 285	astilbin	Sa, Sm	[8,9]
61	9.05	[M−H]	521.1295	521.1295	C_24_H_26_O_13_	521→359, 197	rosmarinic acid-4-*O*-glucoside	Sa	
62	9.38	[M−H]	939.1089	939.1104	C_41_H_32_O_26_	939→769, 617, 393, 317	penta-*O*-galloyl-glucose	P	
63	9.49	[M−H]	515.1184	515.1190	C_25_H_24_O_12_	515→353	dicaffeoylquinic acid	Sa	
64	9.64	[M−H]	449.1088	449.1084	C_21_H_22_O_11_	449→303, 285	neoisoastilbin	Sa, Sm	[8,9]
65	9.68	[M−H]	717.1442	717.1456	C_36_H_30_O_16_	717→519, 475, 321	caffeic acid tetramer	A	
66	9.78	[M−H]	187.0974	187.0970	C_9_H_16_O_4_	187→142, 125	nonandioic acid	P, G	
67	9.84	[M−H]	449.1088	449.1084	C_21_H_22_O_11_	449→303, 285	isoastilbin	Sa, Sm	[8,9]
68	9.87	[M−H]	451.1025	451.1029	C_24_H_20_O_9_	451→341, 299	cinchonain Ib	Sm	
69	9.95	[M−H]	717.1442	717.1456	C_36_H_30_O_16_	717→519, 475, 321	caffeic acid tetramer isomer	A	
70	9.96	[M−H]	597.1605	597.1608	C_30_H_30_O_13_	597→451, 341, 217	glabraoside C	Sa	[8]
71	9.95	[M−H]	719.1599	719.1512	C_36_H_32_O_16_	719→539, 359	dirosmarinic acid	Sa	
72	10.08	[M−H]	433.1131	433.1135	C_21_H_22_O_10_	433→343, 313, 271, 179	(2*R*/2*S*)-naringenin-6-*C*-glucopyranoside	Sa	[8]
73	10.17	[M−H]	433.1131	433.1135	C_21_H_22_O_10_	433→343, 313, 271, 179	(2*R*/2*S*)-naringenin-6-*C*-glucopyranoside	Sa	[8]
74	10.36	[M−H]	587.2327	587.2340	C_27_H_40_O_14_	587→451, 341, 217	sarcaglaboside D	Sa	[8]
75	10.61	[M−H]	515.1184	515.1190	C_25_H_24_O_12_	515→353	dicaffeoylquinic acid	Sa	
76	10.64	[M−H]	359.0768	359.0767	C_18_H_16_O_8_	359→197, 161	rosmarinic acid	Sa, Sm	[8,9]
77	10.73	[M−H]	279.1234	279.1232	C_15_H_20_O_5_	279→235, 139	zedoalactone D	Sa	[8]
78	10.97	[M+COOH]	507.1497	461.1448	C_23_H_26_O_10_	461→417, 295	lactiflorin	P	
79	11.03	[M−H]	433.1132	433.1135	C_21_H_22_O_10_	433→287, 269	engeletin	Sm	[9]
80	11.41	[M−H]	423.1653	423.1655	C_21_H_28_O_9_	423→261, 243	chloranoside A	Sa	[8]
81	11.80	[M−H]	549.1603	549.1608	C_26_H_30_O_13_	549→417, 255	isoliquiritin apioside	G	[26]
82	11.94	[M−H]	433.1132	433.1135	C_21_H_22_O_10_	433→287, 269	isoengeletin	Sa, Sm	[8,9]
83	11.82	[M−H]	549.1607	549.1608	C_26_H_30_O_13_	549→417, 255	liquiritin apioside	G	[26]
84	12.40	[M−H]	417.1183	417.1186	C_21_H_22_O_9_	417→255	isoliquiritin	G	[26]
85	12.97	[M−H]	599.1756	599.1765	C_30_H_32_O_13_	599→569	benzoyloxypaeoniflorin	P	[24]
86	12.98	[M−H]	451.1027	451.1029	C_24_H_20_O_9_	451→341, 299	cinchonain Ic	Sm	[8]
87	13.06	[M−H]	255.0658	255.0657	C_15_H_12_O_4_	255→135	liquiritigenin	G	[26]
88	13.14	[M−H]	451.1025	451.1029	C_24_H_20_O_9_	451→341, 299	cinchonain Id	Sm	[8]
89	13.33	[M−H]	373.0918	373.0923	C_19_H_18_O_8_	373→211, 161	methyl rosmarina	Sa, Sm	[8]
90	13.52	[M−H]	823.4102	823.4116	C_42_H_64_O_16_	823→647, 351	uralsaponin C	G	[27]
91	13.86	[M−H]	835.3742	835.3752	C_42_H_60_O_17_	823→661, 351	uralsaponin D	G	[27]
92	13.52	[M−H]	999.4421	999.4433	C_48_H_72_O_22_	999→837, 645, 351	24-hydroxyl-licorice-saponin A3	G	[27]
93	13.64	[M−H]	895.3950	895.3964	C_44_H_64_O_19_	895→719, 501,351	uralsaponin F	G	[27]
94	13.63	[M−H]	853.3845	853.3858	C_42_H_62_O_18_	853→809, 791, 677, 351	22-hydroxyl-licorice-saponin G2	G	[27]
95	13.61	[M−H]	983.4470	983.4488	C_48_H_72_O_21_	983→821, 645, 351	licorice saponin A3	G	[27]
96	13.68	[M−H]	1025.4579	1025.4593	C_50_H_74_O_22_	1025→993, 833, 497	22-acetoxyl-rhaoglycyrrhizin	G	[27]
97	13.70	[M−H]	849.3538	849.3545	C_42_H_58_O_18_	849→673, 479	uralsaponin E	G	[27]
98	13.76	[M−H]	879.3996	879.4014	C_44_H_64_O_18_	879→861, 643, 351	22-acetoxyl-glycyrrhizin	G	[27]
99	13.77	[M−H]	837.3891	837.3909	C_42_H_62_O_17_	837→819, 661, 351	24-hydroxyl-glycyrrhizin	G	[27]
100	13.78	[M−H]	271.0607	271.0606	C_15_H_12_O_5_	271→254, 177	naringenin	Sm	[27]
101	13.86	[M−H]	835.3742	835.3752	C_42_H_60_O_17_	823→661, 351	24-hydroxyl-licorice-saponin E2	G	[27]
102	14.00	[M−H]	837.3891	837.3909	C_42_H_62_O_17_	837→819, 775, 661, 351	licorice saponin G2	G	[27]
103	14.00	[M−H]	967.4523	967.4539	C_48_H_72_O_20_	967→805,497, 407, 321	rhaoglycyrrhizin	G	[27]
104	14.01	[M−H]	819.3787	819.3803	C_42_H_60_O_16_	819→777, 643, 351	licorice saponin E2	G	[27]
105	14.06	[M−H]	863.4049	863.4065	C_44_H_64_O_17_	863→819, 729, 687, 351, 289	22-acetoxyl-glycyrrhaldehyde	G	[27]
106	14.13	[M−H]	255.0660	255.0657	C_15_H_12_O_4_	255→135	isoliquiritigenin	G	[27]
107	14.14	[M−H]	821.3945	821.3945	C_42_H_62_O_16_	821→803, 759, 645, 351	glycyrrhizin	G	[27]
108	14.33	[M−H]	821.3945	821.3960	C_42_H_62_O_16_	821→803, 759, 645, 351	18α-glycyrrhizin	G	[27]

Note: ^a^: A, *Arnebiae* radix; C, *Curcumae* rhizome; G, *Glycyrrhizae* radix et rhizome; M, *Mume* fructus; P, *Paeoniae* radix rubra; Sa, *Sarcandrae Herba*; Sm, *Smilacis glabrae* rhizome.

### 2.3. Method Validation of the Quantitative Analysis

The calibration curves, linear ranges, LOQ, and repeatability of the 14 analytes were established using the developed UHPLC-MS method (Table 2). Reasonable correlation coefficient values (*r*^2^ > 0.9981) indicated good correlations between the investigated concentrations of the standards and their peak areas within the ranges tested. The ranges of LOQ for all the analytes were from 0.013 to 0.065 μg/mL.

**Table 2:** Calibration curves, linear range, LOQ and repeatability for fourteen compounds analyzed with the UHPLC-MS system.

Analyte	Linear Range (μg/mL)	Calibration Curve (*n* = 7)	*r*^2^	LOQ (μg/mL)	Repeatability RSD (%)
3-*O*-Caffeoylquinic acid (**16**)	0.16–6.37	*y* = 301,311 *x*− 46,304	0.9984	0.064	2.3
5-*O*-Caffeoylquinic acid (**22**)	0.23–9.24	*y* = 341,667 *x*− 39,368	0.9982	0.046	3.2
4-*O*-Caffeoylquinic acid (**24**)	0.41–16.34	*y* = 267,929 *x*− 80,795	0.9990	0.065	3.8
5-*O*-Caffeoylskimic acid (**39**)	0.57–22.92	*y* = 396,547 *x* + 152,855	0.9985	0.057	3.5
Paeoniflorin (**40**)	2.54–101.62	*y* = 207,558 *x* + 1,315,316	0.9988	0.040	1.7
Liquiritin (**51**)	0.46–18.24	*y* = 616,184 *x*+ 109,090	0.9987	0.036	1.8
Neoastilbin (**56**)	0.47–18.84	*y* = 572,723 *x* + 139,006	0.9983	0.016	1.7
Astilbin (**60**)	0.55–22.16	*y* = 500,903 *x* + 165,817	0.9990	0.055	1.5
Neoisoastilbin (**64**)	0.39–15.77	*y* = 339,480 *x* + 93,661	0.9986	0.067	2.0
Isoastilbin (**67**)	0.32–12.84	*y* = 531,497 *x*− 76,292	0.9984	0.018	1.5
Engeletin (**79**)	0.31–12.50	*y* = 743,986 *x*− 120,215	0.9983	0.013	2.1
Isoengeletin (**82**)	0.67–26.66	*y* = 355,937 *x*+ 110,394	0.9987	0.027	3.8
Liquiritigenin (**87**)	0.07–2.96	*y* = 426,840 *x*− 24,980	0.9981	0.030	2.0
glycyrrhizic acid (**107**)	0.99–39.56	*y* = 422,502 *x*+ 468,934	0.9986	0.040	1.6

**Table 3:** Intra-day and inter-day precisions and recoveries for fourteen compounds analyzed with the UHPLC-MS system.

Analyte	Intra-Day (RSD, %) (*n* = 6)	Inter-Day (RSD, %) (*n* = 3)	Recoveries (*n* = 6)				
			Initial (μg)	Spiked (μg)	Detected (μg)	Recoveries (%)	RSD (%)
3-*O*-Caffeoylquinic acid (**16**)	2.9	2.8	1.71	1.75	3.23	92.6	4.8
5-*O*-Caffeoylquinic acid (**22**)	1.3	4.4	1.38	1.27	2.87	108.4	3.8
4-*O*-Caffeoylquinic acid (**24**)	1.3	3.4	2.07	2.25	4.51	104.1	3.4
5-*O*-Caffeoylskimic acid (**39**)	0.6	3.7	2.99	3.15	5.54	90.2	1.4
Paeoniflorin (**40**)	3.2	2.7	27.34	27.95	57.69	104.5	3.8
Liquiritin (**51**)	2.9	2.5	4.95	5.02	10.05	101.3	2.5
Neoastilbin (**56**)	1.9	3.1	5.25	5.18	11.38	109.5	2.3
Astilbin (**60**)	3.4	3.0	6.02	6.10	11.86	97.8	3.7
Neoisoastilbin (**64**)	3.0	3.2	4.24	4.34	8.21	95.6	3.9
Isoastilbin (**67**)	3.2	3.1	3.60	3.53	7.49	105.1	3.2
Engeletin (**79**)	2.5	2.8	3.31	3.44	6.99	104.1	4.1
Isoengeletin (**82**)	3.8	2.3	2.51	2.62	4.83	94.4	3.0
Liquiritigenin (**87**)	2.3	2.6	0.82	0.81	1.52	93.2	2.4
glycyrrhizic acid (**107**)	0.8	1.5	10.75	10.88	22.83	106.0	2.1

The repeatability presented as RSD (*n* = 5) was between 1.5% and 3.8% for the 14 compounds. The overall intra- and inter-day variations (RSD) of the 14 analytes were in the range from 0.6% to 3.8%, and 1.5 to 4.4% (Table 3), respectively. The developed method had good accuracy with the recoveries were between 86.3% and 109.5% (Table 3). Therefore, the results demonstrated that the UHPLC-ESI-MS method was sensitive, precise, and accurate enough for quantitative evaluation of multi-compounds in PSORI-CM01 preparations.

### 2.4. Quantitative Determination of PSORI-CM01 Preparations

A total of eight different batches of PSORI-CM01 preparations were tested using the developed LC-ESI-MS method. The contents (*n* = 3) of 14 investigated compounds are summarized in Table 4. It was recognized that 3-*O*-caffeoylquinic acid (**16**), 5-*O*-caffeoylquinic acid (**22**), 4-*O*-caffeoylquinic acid (**24**), 5-*O*-caffeoylskimic acid (**39**), paeoniflorin (**40**), liquiritin (**51**), neoastilbin (**56**), astilbin (**60**), neoisoastilbin (**64**), isoastilbin (**67**), and glycyrrhizic acid (**107**) were the dominant compounds in all examined samples. However, the contents of each compound or the total content of certain type of constituents varied in different PSORI-CM01 preparation.

**Table 4:** The contents of the 14 compounds in different batches of PSORI-CM01 prepations (*n* = 3).

Analyte ^a^	KLJ-1	KLJ-2	PJ-3	PJ-4	PJ-5	TJ-6	TJ-7	TJ-8
3-*O*-Caffeoylquinic acid (**16**)	520.94	531.48	391.56	132.01	429.07	122.89	30.68	121.18
5-*O*-Caffeoylquinic acid (**22**)	514.55	503.94	391.56	146.51	488.10	123.45	27.30	183.64
4-*O*-Caffeoylquinic acid (**24**)	601.74	545.00	558.82	211.85	683.57	129.75	40.53	160.64
5-*O*-Caffeoylskimic acid (**39**)	540.55	520.10	789.39	324.27	397.06	333.60	153.44	429.46
Paeoniflorin (**40**)	5855.02	6030.52	7218.12	5177.23	5368.14	2326.21	445.51	3511.46
Liquiritin (**51**)	1654.18	1650.73	892.15	360.64	1115.65	145.56	71.55	248.20
Neoastilbin (**56**)	2545.86	2767.29	2442.62	423.15	1680.52	369.88	124.41	588.89
Astilbin (**60**)	3819.23	4061.92	3743.95	844.53	2575.83	661.96	174.14	879.05
Neoisoastilbin (**64**)	2459.06	2359.70	1872.94	736.75	2211.36	605.51	154.36	802.79
Isoastilbin (**67**)	879.60	916.61	650.53	260.87	902.06	244.03	74.96	418.97
Engeletin (**79**)	678.32	740.00	503.10	196.27	621.69	165.13	84.25	274.81
Isoengeletin (**82**)	543.19	1089.55	348.12	132.29	331.67	119.27	16.30	116.39
Liquiritigenin (**87**)	224.07	246.07	477.28	62.14	202.73	75.50	24.55	130.18
glycyrrhizic acid (**107**)	2225.13	2359.89	2610.50	933.51	2770.51	306.01	68.44	257.72

Notes: ^a^ The content unit of granule (KLJ-1, KLJ-2) and pills (PJ-3, PJ-4, PJ-5) was expressed as μg/g; The content unit of decoction was ug/mL.

**Figure 2:**
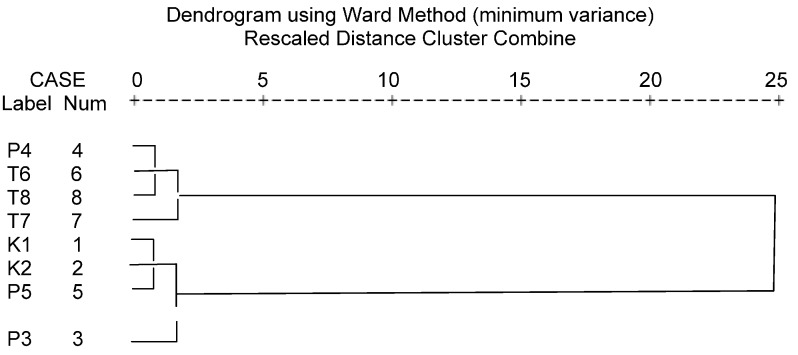
Dendrogram of hierarchical cluster analysis for the eight investigated batches of PSORI-CM01 preparations.

In order to evaluate the variations in detail, hierarchical cluster analysis was performed based on the contents of 14 analytes of the eight investigated batches. Between-groups linkages method was applied, and Squared Euclidean distance was selected as measurement. Figure 2 shows the results on the investigated batches of PSORI-CM01 preparations, which were divided into two main clusters. The results suggested that the contents of 14 analytes were relatively more stable and higher in granule preparations (the batches of K1 and K2) but varied in tablet and decoction preparations. This may be related to the origin of raw medicinal plants origin and extraction technology and so on. As mentioned above, 3-*O*-caffeoylquinic acid (**16**), paeoniflorin (**40**), liquiritin (**51**), astilbin (**60**), engeletin (**79**), liquiritigenin (**87**) and glycyrrhizic acid (**107**) may be the main active components of PSORI-CM01, The content of the six ingredients should be stressed on in establishing quality control methods for PSORI-CM01 preparations.

## 3. Experimental Section

### 3.1. Chemicals and Materials

Methanol and acetonitrile (HPLC grade) were purchased from Fisher Scientific (Fair Lawn, NJ, USA); Formic acid (HPLC grade) was purchased from the Sigma-Aldrich (Seelze, Germany); Ultra-pure water was prepared using a Millipore Milli-Q purification system (Bedford, MA, USA).

5-*O*-caffeoylskimic acid (**39**), paeoniflorin (**40**), neoastilbin (**56**), astilbin (**60**), neoisoastilbin (**64**), isoastilbin (**67**), engeletin (**79**) and isoengeletin (**82**) were isolated in our lab and identified by the authors. 3-*O*-caffeoylquinic acid (**16**), 5-*O*-caffeoylquinic acid (**22**), 4-*O*-caffeoylquinic acid (**24**), liquiritin (**51**), liquiritigenin (**87**), and glycyrrhizic acid (**107**) were purchase from the National Institutes for Food and Drug Control (Beijing, China). All of the purities were above 98% by HPLC analysis. Eight batches of PSORI-CM01 preparations were supplied by Guangdong Provincial Hospital of Chinese Medicine, and voucher samples were deposited in the laboratory of Materia Medica Preparation, Guangdong Province Academy of Chinese Medicine Science.

### 3.2. Standard Solutions and Sample Preparation

Stock solutions of the 14 reference substances were prepared in concentrations ranging from 0.420 to 5.083 mg/mL in 60% methanol and stored at 4 °C until use. A standard working solution of the mixtures was obtained by diluting stock solutions to desired concentrations. Aliquots of this solution were further diluted with initial mobile phase to a series of concentrations for quantification.

PSORI-CM01 preparations (granules and tablets) were pulverized into a fine powder. The powder (0.5 g) was accurately weighed, immersed in 60% MeOH (v/v, 10 mL) for 1 h at room temperature, then extracted in an ultrasonic water bath for 30 min. After recording the weight, the extract was filtered through filter paper. Aliquots of 500 μL of filtrate was transferred into a 5 mL volumetric flask which was made up to its volume with initial mobile phase. PSORI-CM01 decoction was diluted 10 times in 60% MeOH (v/v) for quantitative analysis. All of the samples were filtered through a 0.22 μm syringe filter before use, and 10 μL was injected into the LC instrument for LC-MS analysis.

### 3.3. UHPLC-ESI-MS/MS System

Chromatographic separation was performed on an Accela™ ultra high pressure liquid chromatography (UHPLC) system (Thermo Fisher Scientific, San Jose, CA, USA) comprising a UHPLC pump, a PDA detector, scanning from 200 to 400 nm, and an autosampler settled to 30 °C. The LC conditions were as follows: column: Thermo Scientific Syncronis C18 (50 mm × 2.1 mm, 1.7 μm); mobile phase: acetonitrile (A) and water containing 0.1% (v/v) formic acid (B); gradient: 0 min, 5:95; 10 min, 27:83; 15 min, 50:50; 18–20 min, 100:0 (A:B, v/v); flow rate: 0.4 mL/min; injection volume: 10 μL.

The above UHPLC system was connected with a LTQ/Orbitrap mass spectrometry system (Thermo-Fisher Scientific, Bremen, Germany) via an ESI interface. High purity nitrogen (N_2_) was used as the sheath gas and helium (He) as the auxiliary gas with a flow rate of 40 and 10 arbitrary units, respectively.

### 3.4. Qualitative Characteristic of Chemical Constituents

Identification of chemical constituents in PSORI-CM01 preparations was performed by LC-ESI-MS^n^ analysis. The ESI-MS spectra of samples and reference compounds were acquired in negative ionization mode. The parameters were as follows: capillary temperature at 320 °C, capillary voltage at −28 V, ion spray voltage at −4.0 kV, and tube lens voltage at −90 V. For full scan MS analysis, the spectra were recorded in the range of *m/z* 100–1500 with a resolution of 30,000. Data-dependant acquisition was applied and the most intense ions detected in each MS scan were selected for MS^n^ data records with a resolution of 15,000. The activation time was 30 ms and the collision energy was adjusted to 35%. Data were processed by Xcalibur software (Thermo-Fisher Scientific, Bremen, Germany). An external calibration for mass accuracy was carried out the day before the analysis according to the manufacturer’s guidelines.

### 3.5. Validation of the Quantitative Analysis

The stock solution containing 14 reference compounds was prepared and diluted to seven-point calibration levels for the construction of calibration curves. Each concentration of the mixed standard solution was injected in triplicate. Calibration curves were established by plotting the peak area *versus* concentration of each analyte. Intra- and inter-day variations were utilized to assess the precision of the method. The intra-day variation was determined by analyzing six replicates within 1 day and the inter-day variation was examined in three consecutive days. Recovery was used to evaluate the accuracy of the method. Recovery test was carried out to investigate accuracy of this method by adding certain amounts of the 14 standard solutions to 0.25 g powder of sample in sextuplicate. Samples were processed as described in Section 2.2. To confirm the repeatability, five replicates of the same sample were extracted and analyzed. Variations were expressed by relative standard deviation (RSD) in all three tests above.

## 4. Conclusions

An efficient and sensitive method employing ultra-high liquid chromatography coupled with linear trap quadrupole and high resolution mass analyzer-orbitrap (UHPLC-LTQ/Orbitrap) was developed for the qualitative and quantitative analysis of chemical constituents of PSORI-CM01 preparations. One hundred and eight compounds, including organic acids, phenolic acids, flavonoids, and terpenoids, were characterized on the basis of retention behaviors, abundant MS and MS^n^ data, or by comparing with reference substances and literatures. All compounds identified were found to be existed in individual traditional Chinese medicines of PSORI-CM01 preparation. However, the characteristic constituents from *Rhizoma Curcumae* were not detected. Further investigation focused on those lipophilic constituents in PSORI-CM01 preparation is required.

An optimized LC-ESI-MS method was then established for assay of the 14 marker compounds in PSORI-CM01 preparations. The validation of the method, represent a good accuracy, sensitivity and repeatability. The quantification results indicated an obvious difference of marker compounds contents among various samples. This is the first report on the comprehensive determination of chemical constituents in PSORI-CM01 preparations by LC-ESI-MS^n^. The results would provide the chemical support for the further pharmacokinetic studies and for the improvement of quality control of PSORI-CM01 preparations. The study also suggested that LC-LTQ/Orbitrap-MS would be a powerful and reliable analytical tool for the characterization of chemical profile in complex chemical system, such as TCM preparations.
